# Spatial and temporal (non)binding of audiovisual rhythms in sensorimotor synchronisation

**DOI:** 10.1007/s00221-023-06569-x

**Published:** 2023-02-14

**Authors:** Olivia Morgan Lapenta, Peter E. Keller, Sylvie Nozaradan, Manuel Varlet

**Affiliations:** 1 The MARCS Institute for Brain, Behaviour and Development, Western Sydney University, Penrith, Australia; 2 Present Address: Psychological Neuroscience Lab, Center for Investigation in Psychology, University of Minho, Rua da Universidade, 4710-057 Braga, Portugal; 3 Center for Music in the Brain, Department of Clinical Medicine, Aarhus University, Aarhus, Denmark; 4 Institute of Neuroscience, Université Catholique de Louvain, Woluwe-Saint-Lambert, Belgium; 5 School of Psychology, Western Sydney University, Penrith, Australia

**Keywords:** Frequency tagging, Motor tracking, Multisensory integration, Movement synchronisation, Steady-state evoked potentials

## Abstract

Human movement synchronisation with moving objects strongly relies on visual input. However, auditory information also plays an important role, since real environments are intrinsically multimodal. We used electroencephalography (EEG) frequency tagging to investigate the selective neural processing and integration of visual and auditory information during motor tracking and tested the effects of spatial and temporal congruency between audiovisual modalities. EEG was recorded while participants tracked with their index finger a red flickering (rate *f*_V_ = 15 Hz) dot oscillating horizontally on a screen. The simultaneous auditory stimulus was modulated in pitch (rate *f*_A_ = 32 Hz) and lateralised between left and right audio channels to induce perception of a periodic displacement of the sound source. Audiovisual congruency was manipulated in terms of space in Experiment 1 (no motion, same direction or opposite direction), and timing in Experiment 2 (no delay, medium delay or large delay). For both experiments, significant EEG responses were elicited at *f*_V_ and *f*_A_ tagging frequencies. It was also hypothesised that intermodulation products corresponding to the nonlinear integration of visual and auditory stimuli at frequencies *f*_V_ ± *f*_A_ would be elicited, due to audiovisual integration, especially in Congruent conditions_._ However, these components were not observed. Moreover, synchronisation and EEG results were not influenced by congruency manipulations, which invites further exploration of the conditions which may modulate audiovisual processing and the motor tracking of moving objects.

## Introduction

The integration of multiple sensory modalities, such as visual and auditory inputs, is critical to promote adequate perception and behavioural responses in dynamic environments, as these signals often arise from common external events or objects (Macaluso and Driver 2005). The combination of co-occurring and co-localised visual and auditory cues can lead to enhanced processing of visual signals (Frassinetti et al. 2002). Importantly, such integration strongly relies on temporal and spatial congruency. Huddleston and colleagues (2008) revealed that the perception of continuous rotational stimuli observed in unimodal auditory and visual conditions vanishes in bimodal conditions where visual vertical and auditory horizontal stimuli are intercalated, showing that spatiotemporal congruence is needed for effective multimodal integration and perception.

Co-occurring and co-localised audiovisual (AV) stimuli are not only beneficial for perception, but also in motor coordination tasks, including motor coordination with a moving object (Meyer et al. 2005) or another individual (Noy et al. 2017). In particular, Rosati and colleagues (2012) investigated the role of different types of auditory feedback in motor learning and motor control, as additional sensory information to visual and proprioceptive modalities. They found that auditory feedback simulating the sound of a rolling ball improved performance in visual tracking of a dot moving horizontally on the screen compared to conditions without sound feedback or error-related sound feedback. Thus, adding auditory information enhances perception of task-relevant information and improves sensory-motor coordination only when auditory information is congruent with visual information.

Investigations with neuroimaging techniques have been employed to uncover the brain structures and mechanisms underlying AV integration in motion perception and motor coordination. Alink et al. (2012) identified two areas encoding auditory motion stimuli, specifically the planum temporale as a key area for auditory motion perception and high-level visual cortex, suggesting a convergence of motion signals from both visual and auditory modalities. Von Saldern and Noppeney (2013) likewise found an important role of visual areas (hMT + /V5 +) together with the planum temporale in the processing of auditory motion stimuli. They also revealed stronger activation of the putamen in audiovisual conditions, especially when stimuli were degraded and a bimodal advantage on motion discrimination accuracy was observed, which suggests that bimodal integration is favoured under challenging conditions.

Neuroimaging investigations in motor coordination tasks have been largely limited due to movement restrictions and artefacts, leaving the underlying audiovisual integration processes and the effects of spatial and/or temporal congruency unclear. However, growing evidence suggests EEG frequency tagging (i.e. steady-state evoked potentials (SSEPs)) as a promising method for addressing these questions (Gordon et al. 2019). By tagging sources of information in the environment at specific frequencies, their neural processing and integration can be reliably examined even if participants are moving (Nozaradan et al. 2015; Varlet et al. 2020). Neural populations involved in the processing of a tagged stimulus exhibit increased activity at this specific frequency (Gordon et al. 2019). SSEPs therefore offer the possibility to tag multiple stimuli, and disentangle their respective processing in EEG signals based on their tagging frequencies (Norcia et al. 2015; Renton et al. 2018; Varlet et al. 2020). Further, frequency tagging enables the investigation of the neural integration of two different sources of information, as reflected in increased amplitude at the intermodulation frequency components, corresponding to the addition or subtraction of the two fundamental tagging frequencies and their harmonics (Gordon et al. 2019).

However, while neural responses at the intermodulation frequencies have been shown within sensory modalities (e.g. between two visual stimuli) in numerous studies, evidence supporting neural responses at the intermodulation frequencies across sensory modalities is still lacking. Indeed, Giani et al. (2012) compared passive perception under uni- and bimodal conditions, and found responses at the intermodulation frequencies only in the unimodal visual condition but not in the bimodal audiovisual condition. In follow-up experiments using a detection task, the authors investigated if actively attending to either auditory or visual stimuli promote integration, and again they failed to identify amplitude increases at the intermodulation frequencies. Interestingly, in a study addressing the coupling between auditory and motor responses during sensorimotor synchronisation with beat sequences, Nozaradan and colleagues found activity at the intermodulation frequencies corresponding to the sum of the auditory beat frequency and participant’s movement frequency while tapping a finger in time with every second beat (Nozaradan et al. 2015). These findings highlight the need for further investigations to better understand the relevance of intermodulation frequencies for understanding integration across sensory modalities.

Here, we investigated the relevance of EEG frequency tagging to better understand the neural processing of visual and auditory information and their integration underpinning effective motor synchronisation with a continuously moving stimulus, and determine how these processes are modulated by spatial and temporal (in)congruency between the two modalities. We conducted two experiments to address these questions in which we examined the motor tracking performance of a visual stimulus oscillating horizontally on a monitor with a complex movement trajectory while manipulating either the temporal or spatial congruency of an auditory stimulus with respect to the moving visual stimulus. Visual and auditory stimuli were tagged at specific frequencies (*f*_V_ = 15 Hz and *f*_A_ = 32 Hz, respectively) to examine the underlying neural tracking and integration of visual and auditory information and how they are modulated by temporal and spatial congruence. We hypothesised significant EEG responses at *f*_V_ and *f*_A_ tagging frequencies that would be greater in the Congruent conditions since temporal congruency has been shown to enhance the processing of multisensory inputs at sensory-specific stages of cortical processing (Nozaradan et al. 2012) and considering that spatially and temporally Incongruent conditions are likely to promote divided attention (De Jong et al. 2010). Further, significant amplitude at the intermodulation frequencies (*f*_V_ ± *f*_A_—17 and/or 47 Hz) was expected in EEG recordings as signature of neural integration of visual and auditory information, especially in temporally and spatially Congruent conditions and in visual areas (Alink et al. 2012; von Saldern and Noppeney 2013; Giani et al. 2012).

## Materials and methods

### Participants

We invited 19 subjects to participate in the spatial sound manipulation experiment (Experiment 1—E1) and 19 to participate in the temporal sound manipulation experiment (Experiment 2—E2). The sample size was chosen based on an a priori power analysis with G-Power (V. 3.1.9.3) to detect medium effect sizes (*f* = 0.25) with at least 80% power, in line with previous similar studies (Giani et al. 2012). The experiments were conducted in accordance to the Declaration of Helsinki and approved by the Human Research Ethics Committee at Western Sydney University (ethics reference number: H13092). All participants gave their written informed consent and complied with the following criteria: age between 18 and 50 years, right-handed, normal or corrected-to-normal visual acuity, and no known past or current auditory impairment, psychological or psychiatric disorders, and central nervous system injury. One participant in each experiment was excluded due to EEG recordings with abnormal noise. Therefore, the final sample of E1 involved 18 participants (13 female) aged between 18 and 42 years (*M* = 25.50; SD = 6.82) and of E2 involved 18 participants (11 female) aged between 18 and 42 years (*M* = 25.66; SD = 6.83).

### EEG and motion-tracking recordings

Electrophysiological (EEG) data were recorded with a BioSemi ActiveTwo system (BioSemi, The Netherlands) by means of 64 active Ag–AgCl electrodes placed over the scalp according to the International 10/20 system. Additionally, two external Ag–AgCl electromyography (EMG) electrodes were positioned on the corner and bottom of the left eye to record blinks and eye movements. EEG and EMG were recorded at a sampling rate of 2048 Hz, and stored for off-line analyses.

Motion tracking was used to record the horizontal oscillations of participant’s right index finger at a sampling rate of 240 Hz with 0.01 mm spatial resolution via a Polhemus LIBERTY motion tracker (Polhemus Ltd., VT, USA). The movement data were used in real time to control the horizontal position of the cursor on the monitor and saved on a PC for off-line analyses of the synchronisation performance.

### Stimuli and procedure

Auditory stimuli were pure tones (sinusoidal waveform) with lateralised amplitude modulations in left and right audio channels (panning) presented via insert earphones (ER‐1, Etymotic Research, Elk Grove Village, IL, USA). The auditory stimuli were tagged using continuous (sinusoidal) pitch modulation at 32 Hz between 500 and 1100 Hz. In other words, the carrier frequency of the sound continuously and sinusoidally alternated between 500 and 1100 Hz at 32 Hz. We opted to modulate the pitch because sound amplitude modulation was already used to generate the panning (i.e. lateralised modulation of amplitude in the left and right audio channels) to create the perception of motion. Comfortable intensity at approximately 70 dB kept the same for all participants was used.

The moving target and the participant's cursor were red (RGB: 255,0,0) dots with 3.2 cm of diameter presented on a 24-inch VIEWPixx LCD monitor (VPixx Technologies, Saint-Bruno, Canada) with a 120 Hz refresh rate. Participants were seated approximately 60 cm from the monitor. The target visual stimuli flickered at 15 Hz and moved horizontally across the screen with a complex movement trajectory that changed every trial to make it more difficult for the participant to track. The target horizontal movement corresponded to the sum of three sine waves (1: 0.185 Hz and 14.5 cm; 2: 0.74 Hz and 9.7 cm; and 3: 1.11 Hz and 4.8 cm, of frequency and amplitude, respectively) with new random phase values for each trial. The participant’s cursor was positioned 2 cm below the target, had no flickering, and its position was controlled in real time using motion-capture data.

Although visual SSEPs can be recorded in several ranges, usually studies opt for frequencies within 3–20 Hz with most of them using frequencies above 8–10 Hz (Norcia et al. 2015). We chose to present the visual stimuli at 15 Hz to avoid interference in alpha frequencies and lower frequencies due to the movements of the participants and the moving stimuli. 32 Hz for the auditory stimulus was selected to ensure that auditory fundamental and audiovisual intermodulation frequencies were also above movement and alpha frequencies while avoiding overlaps between their harmonics.

**Fig. 1:**
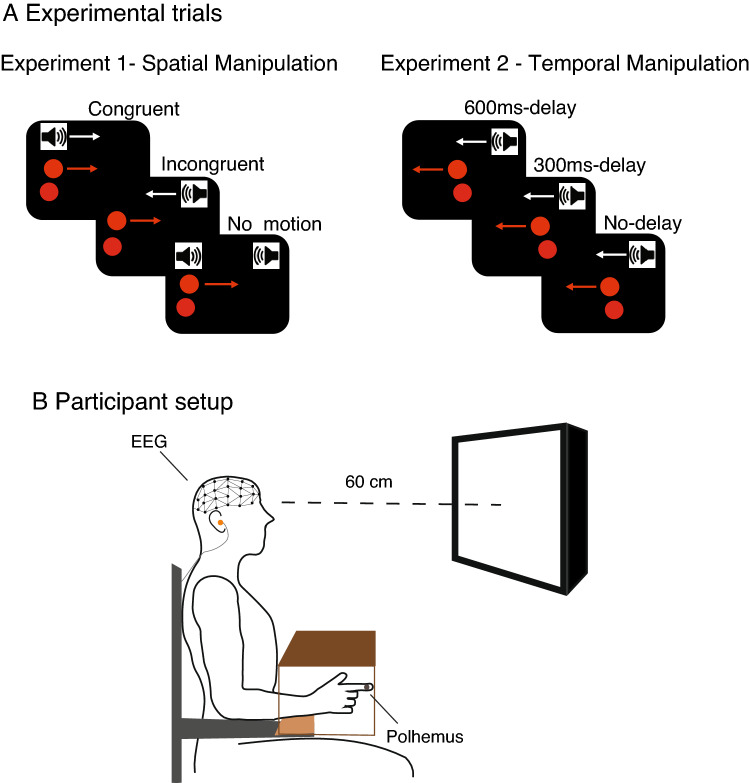
HYPERLINK "sps:id::fig1||locator::gr1||mediaobject::0" Experimental design. Panel **A** Schematic illustration of the different experimental conditions, the arrows illustrate the movement of auditory and visual stimuli, and the upper red dot illustrates the visual stimuli, whereas the bottom one represents the visual feedback of the motion tracker. Panel **B** The experimental setup

Audiovisual congruency was manipulated to generate three conditions for each experiment. For E1, we manipulated spatial congruency; specifically, sound would either (i) move congruently with the visual target by lowering the volume in the ear on the *opposite* side of the moving direction of the visual target while increasing the volume in the ear on the *same* side (Congruent), (ii) move incongruently with the target by lowering the volume in the ear on the *same* side of the moving direction of the visual target while increasing the volume in the ear on the *opposite* side, i.e. in antiphase (Incongruent), or (iii) stay at consistent volume in both ears, with no perception of auditory motion (No-motion). Combined amplitude (volume) across the two ears was the same for the three conditions. For E2, we manipulated time congruency; specifically, sound would either (i) move simultaneously with the visual target (No-delay); (ii) move with a time delay of 300 ms (300 ms delay); or (iii) move with a time delay of 600 ms (600 ms delay) (Fig. 1A).

Upon arrival, all participants were informed about the procedure and provided written informed consent. After EEG preparation, participants were asked to seat comfortably on a chair with their right hand positioned on a wooden board over their lap. The motion sensor was attached to the participant’s right index finger, which was occluded from sight using a custom-built cardboard box (Fig. 1B). Participants then received the verbal and on-screen instruction to do their best to track the horizontal displacements of the visual stimulus with the cursor on the screen that was controlled in real time by the horizontal oscillations of their index finger. Each participant was allocated either in E1 or E2. Initially, they performed practice trials in which one sample trial of each condition was presented, and thus a total of three practice trials. These practice trials allowed participants to familiarise to the use of the tracking sensor and the control of their cursor on the screen. After these practice trials, the experimenter made sure that participants understood the task and were performing accordingly, and the actual experiment began.

Participants were presented with a total of 30 trials with a duration of 60 s, including 10 trials for each audiovisual combination. Ten different complex stimulus trajectories were used, each presented once in a randomised order for each of the three sound conditions of E1 and E2. At the completion of each trial, a neutral screen appeared informing the participant of the trial number, and again displaying the instruction to follow the target with the cursor. Participants set their own pace regarding trial progression, as they pressed the space bar on a keyboard situated beneath the monitor in front of them to continue to the next trial. A break was given halfway through the trials. At the completion of the 33 trials (3 practice + 30 experimental), participants had all sensors and electrodes removed and were then debriefed regarding the experiment. Participants took roughly 40 min to complete the task and the EEG preparation varied between 30 and 50 min. Therefore, the experiment was in total about 80 min long.

### EEG data processing

Data were processed using Fieldtrip toolbox for EEG analysis (Oostenvend et al. Oostenveld et al. 2011) in the MatLab software environment (The MathWorks, Natick, USA). When more than 50% of trials was contaminated with excessive EEG and/or EMG noise, the participant was excluded (*n*_E1_ = 1; *n*_E2_ = 1). For the remaining participants (18 in each experiment), channels with excessive noise were interpolated by the mean of surrounding channels; a maximum of three and of five EEG channels per participant was interpolated in Experiment 1 (*M*_E1_ = 1; SD_E1_ = 0.88) and Experiment 2 (*M*_E2_ = 1.27; SD_E2_ = 1.28), respectively. Trials were considered bad trials and rejected when visual inspection detected large waves in EEG due to loss of reference or ground channels. Each participant had maximum three rejected trials (*M*_E1_ = 0.27; SD_E1_ = 0.8; *M*_E2_ = 0.22; SD_E2_ = 0.71) due to poor EEG quality. Independent component analysis was performed using FASTICA algorithm, as implemented in Fieldtrip, to identify and reject eye blink artefacts. The average number of ICA components removed was *M*_E1_ = 1.6 (SD_E1_ = 0.61) and *M*_E2_ = 1.94 (SD_E2_ = 0.85). A 0.1 Hz high-pass filter was applied and data were notched filtered to remove 50 Hz power contamination and its harmonics. EEG data were re-referenced to the average of all EEG channels. Data were downsampled to 1000 Hz and stored for further analyses.

For each participant and condition, EEG epochs were averaged across trials in the time domain. Across-trial averaging in the time domain is expected to cancel out or at least markedly reduce the contribution of EEG signals that are not phase locked to the stimulation train, and therefore increase signal-to-noise ratio (Mouraux and Iannetti 2008). Subsequently, for each participant and condition, the obtained waveforms were then examined in the frequency domain by using a discrete Fourier transform (Frigo and Johnson 1998), using a Hanning window, yielding a power spectrum ranging from 0 to 100 Hz, since the spectrum was trimmed up to 100 Hz, with a frequency resolution of 0.017 Hz (i.e. 1/60).

**Fig. 2:**
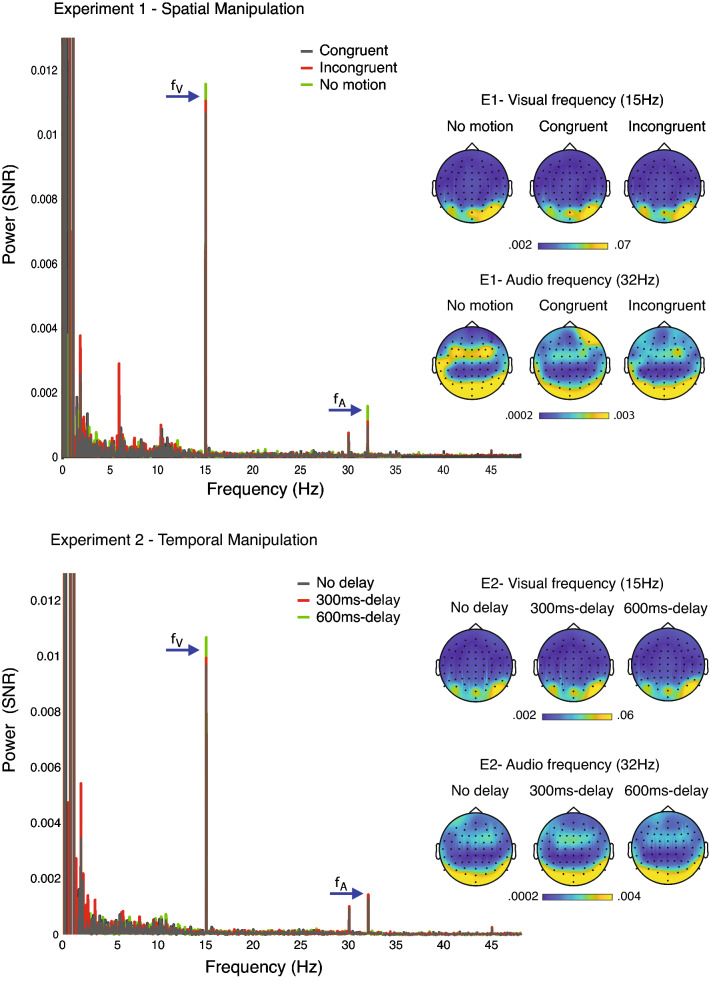
Power as a function of frequency and topopolots of the target frequencies. Baseline-subtracted power spectra and topographic plots were generated based on the average of all electrodes. The blue arrows indicate the fundamental frequencies of interest for the visual (*f*_V_ = 15 Hz) and auditory (*f*_A_ = 32 Hz) stimuli. Upper and lower panels show the averaged data of all participants for Experiment 1 and 2, respectively

To remove unrelated background noise due to spontaneous EEG activity, muscle activity or eye movements, and obtain estimates of the magnitude of the auditory and visual steady-state evoked potentials (SSEPs) and their intermodulation frequencies, data were normalised by performing a baseline subtraction. Noise was removed by subtracting, at each bin of the frequency spectra, the average power measured at ten neighbouring frequency bins excluding adjacent ones (Varlet et al. 2020; Lenc et al. 2018), with bin size determined by the frequency resolution of the Fourier transform. Finally, the neural tracking and integration of the visual and auditory stimuli were estimated by averaging the power of all EEG channels at the frequencies corresponding to visual (15 Hz) and auditory (32 Hz) tagging frequencies and their intermodulation frequencies (17 and 47 Hz). Topographic maps were generated to visualise the distribution of the EEG responses at the frequencies of interest (Fig. 2).

### Statistical analysis

All statistical analyses were carried out using JASP software (JASP Team 2018, https://jasp-stats.org/) with alpha = 5% for analysis of variance and pairwise comparisons. Further, Bayesian equivalent tests were performed to report statistical evidence using Bayes factors (BFs), BF_10_ for paired sample comparisons and correlational analysis and BF_incl_ for ANOVAs denoting the level of evidence for the alternate hypothesis (non-signed difference), and the inclusion of a specific parameter in a model (ANOVA), respectively.

#### Behaviour

For each participant and condition, we computed the mean distance between the visual target and the participant’s cursor. We then conducted for each experiment a one-way repeated-measures ANOVA considering Condition as a within-subject factor.

#### SSEPs

For each experiment, we conducted a one-way repeated-measures ANOVA considering Condition as a within-subject factor on the baseline-subtracted power at each frequency of interest, i.e. 15 Hz for visual and 32 Hz for auditory stimuli, and the intermodulation frequencies representing their integration (i.e. 17 and 47 Hz).

Furthermore, to examine the occurrence of significant EEG responses at the four frequencies of interest, we averaged the power spectra of all EEG channels together before baseline subtraction and computed *Z*-scores at each frequency bin as the difference in power between that frequency bin and the mean of the ten neighbouring frequency bins (excluding the two immediately adjacent frequency bins), divided by the standard deviation of those ten neighbouring bins. *Z*-scores were computed individually and at the group level (power spectra averaged across participants) for each condition and averaged between conditions. EEG responses at specific frequency bins were considered to be significant when the *Z*-score value was greater than 3.1 (*p* < 0.001, one-tailed), in line with previous studies that used frequency tagging techniques (Varlet et al. 2020; Jacques et al. 2016; Quek et al. 2018), which indicated signal power significantly greater than the noise background.

#### Correlation between behavioural and SSEPs data

Finally, we tested whether changes in EEG and behavioural responses across the different conditions were correlated, i.e. whether increase in SSEP power was accompanied by better motion-tracking performance. Therefore, we computed for each experiment EEG and behavioural changes relative to the control audio condition. For both mean distance and SSEP power, we subtracted the control condition (i.e. no motion in E1 and No-delay in E2) from the other conditions (i.e. Congruent and Incongruent in E1, and 300 ms and 600 ms delay in E2) and performed Pearson correlation analyses between the two for each frequency of interest.

## Results

### Motion-tracking performance

#### Experiment 1

**Fig. 3:**
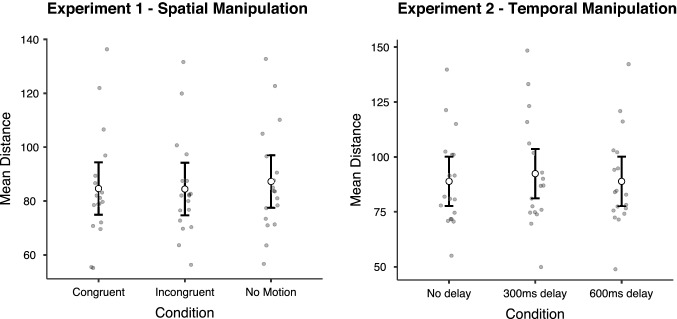
Motion-tracking results. Graphs depict group and individual mean distance with bars representing the confidence interval between the visual target and the motion tracker cursor in each experimental condition for both experiments

The rmANOVA performed for the mean distance, with Greenhouse–Geisser correction because sphericity assumption was violated (*x*^2(2)^ = 7.573, *p* = 0.023), revealed no significant effect for condition (*F*_1,17_ = 3.458; *p* = 0.061; *η*_p_^2^ = 0.169, BF_Incl_ = 1.500), as depicted in Fig. 3.

#### Experiment 2

The rmANOVA performed for the mean distance, with Greenhouse–Geisser correction because sphericity assumption was violated (*x*^2(2)^ = 13.878 *p* = 0.001), revealed no significant effect for condition (*F*_1,17_ = 2.823; *p* = 0.100; *η*_p_^2^ = 0.142, BF_Incl_ = 0.995), as depicted in Fig. 3.

### SSEPs

#### Experiment 1

##### Fundamental frequencies

Reliable peaks at the fundamental frequencies (i.e. 15 and 32 Hz) based on *Z*-score data were observed at group level in all conditions. They were also reliably observed at individual level. Significant peaks at 15 Hz for the visual tagging were observed in all conditions and all participants. Significant peaks at 32 Hz for the audio tagging were found in 13, 15 and 15 over 18 participants in the Congruent, Incongruent and No motion conditions, respectively.

**Fig. 4:**
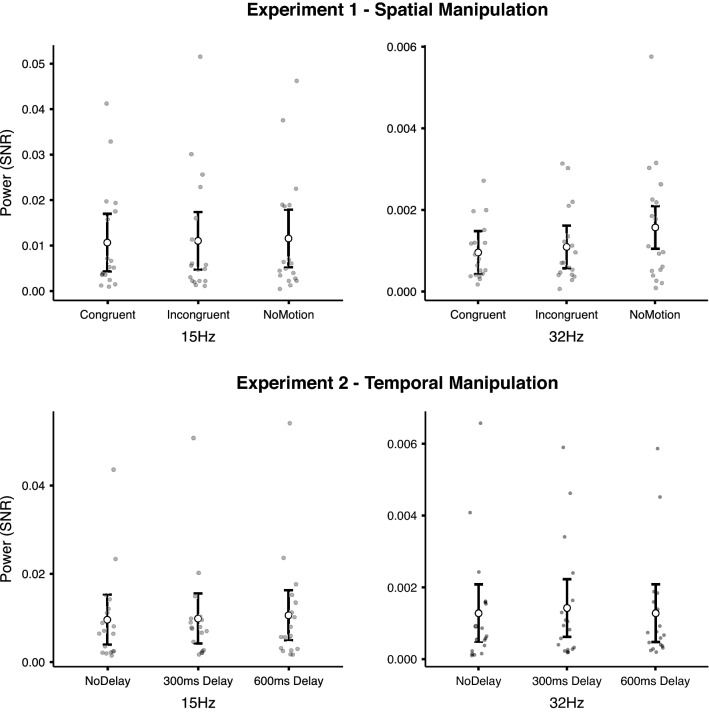
Target frequencies across conditions. Graphs depict group and individual signal-to-noise ratio of the power spectra generated based on the average of all electrodes with bars representing the confidence interval

The rmANOVA performed on the SNR of EEG power at 15 Hz revealed no significant effect for condition (*F*_1,17_ = 0.794; *p* = 0.460; *η*_p_^2^ = 0.045, BF_Incl_ = 0.251). The rmANOVA on the EEG power at 32 Hz with Greenhouse–Geisser correction, because sphericity assumption was violated (*x*^2(2)^ = 7.901, *p* = 0.019), revealed a significant effect of condition (*F*_1,17_ = 7.320; *p* = 0.007; *η*_p_^2^ = 0.301, BF_Incl_ = 15.704). Bonferroni corrected pairwise comparisons revealed significant differences between the No motion condition when compared to Congruent (*t*(17) = 2.924, *p* = 0.009, *d* = 0.689, BF_10_ = 5.528) and Incongruent (*t*(17) = 2.870, *p* = 0.011, *d* = 0.676, BF_10_ = 5.028), but no differences between Congruent and Incongruent conditions (*t*(17) = 1.171, *p* = 0.258, *d* = 0.276, BF_10_ = 0.440). This result suggests larger EEG response to the audio stimulus in the No motion condition compared to the Congruent and Incongruent conditions. Grand-average power spectra for each condition are presented in Fig. 2 and EEG power at 15 and 32 Hz more specifically with individual participants’ data points are presented in Fig. 4.

##### Intermodulation frequencies

Analyses on *Z*-score data did not show significant peaks at the intermodulation frequencies at the group level in any conditions and at the individual level in any participants and conditions. The rmANOVA revealed no significant effect of condition for 17 Hz (*F*_1,17_ = 0.197; *p* = 0.822; *η*_p_^2^ = 0.011, BF_Incl_ = 0.173) or for 47 Hz (*F*_1,17_ = 1.371; *p* = 0.267; *η*_p_^2^ = 0.075, BF_Incl_ = 0.442).

Analyses (not presented here) conducted on the harmonics of the fundamental and intermodulation frequencies, which can also contain relevant activity despite much smaller magnitude, did not show any significant effects either (all *p* values > 0.05).

#### Experiment 2

##### Fundamental frequencies

*Z*-score data indicated reliable peaks at the fundamental frequencies (i.e. 15 and 32 Hz) at the group level in all conditions. At the individual level, significant peaks at 15 Hz for the visual tagging were observed in all conditions and all participants, and significant peaks at 32 Hz for the audio tagging were found in 12, 13 and 13 over 18 participants in No-delay, 300 ms delay and 600 ms-delay conditions, respectively.

The rmANOVA performed on the EEG power at 15 Hz revealed no significant effect for condition (*F*_1,17_ = 1.222; *p* = 0.307; *η*_p_^2^ = 0.067, BF_Incl_ = 0.346). The rmANOVA on the EEG power at 32 Hz also showed no significant effect of condition (*F*_1,17_ = 1.463; *p* = 0.246; *η*_p_^2^ = 0.079, BF_Incl_ = 0.405). Grand-average power spectra for each condition are presented in Fig. 2 and EEG power at 15 Hz and 32 Hz, in particular with individual participants’ data points are presented in Fig. 4.

##### Intermodulation frequencies

*Z*-score analyses showed no significant peaks at the intermodulation frequencies at group level in any conditions and at individual level in any participants and conditions. The rmANOVA conducted on the intermodulation frequencies revealed no significant effect of condition for 17 Hz (*F*_1,17_ = 1.698; *p* = 0.198; *η*_p_^2^ = 0.091, BF_Incl_ = 0.482) or for 47 Hz (*F*_1,17_ = 0.707; *p* = 0.500; *η*_p_^2^ = 0.040, BF_Incl_ = 0.272).

### Correlation between SSEPs and motion-tracking performance

#### Experiment 1

Correlational analyses on the changes in EEG power and motion-tracking performance relative to the control condition indicated no significant correlations between motion-tracking performance and EEG power at the 15 Hz visual tagging frequency in the Congruent condition (*r* = 0.007, *p* = 0.978, BF_10_ = 0.291) and the Incongruent condition (*r* = 0.137, *p* = 0.587, BF_10_ = 0.334) or EEG power at the 32 Hz audio tagging frequency in the Congruent condition (*r *= −0.151, *p* = 0.549, BF_10_ = 0.344) and the Incongruent condition (*r* =  − 0.189, *p* = 0.453, BF_10_ =  − 0.189). Correlational analyses indicated no significant correlations between motion-tracking performance and EEG power at the 17 Hz intermodulation frequency in the Congruent condition (*r* =  − 0.264, *p* = 0.289, BF_10_ = 0.491) and the Incongruent condition (*r* = 0.050, *p* = 0.844, BF_10_ = 0.297), and between motion-tracking performance and EEG power at the 47 Hz intermodulation frequency in the Congruent condition (*r* = 0.184, *p* = 0.464, BF_10_ = 0.374) and the Incongruent condition (*r* = 198, *p* = 0.431, BF_10_ = 0.389).

#### Experiment 2

Correlational analyses indicated no significant correlations between motion-tracking performance and EEG power at the 15 Hz visual tagging frequency in the 300 ms-delay condition (*r* = −0.279, *p* = 0.262, BF_10_ = 0.523) and the 600 ms-delay condition (*r* = 0.224, *p* = 0.371, BF_10_ = 0.423) or EEG power at the 32 Hz audio tagging frequency in the 300 ms-delay condition (*r* = −0.107, *p* = 0.672, BF_10_ = 0.317) and the 600 ms-delay condition (*r* = −0.138, *p* = 0.585, BF_10_ = 0.335). Correlational analyses indicated no significant correlations neither between motion-tracking performance and EEG power at the 17 Hz intermodulation frequency in the 300 ms-delay condition (*r * = −0.290, *p* = 0.243, BF_10_ = 0.550) and the 600 ms-delay condition (*r* = 0.237, *p* = 0.237, BF_10_ = 0.442), and between motion-tracking performance and EEG power at the 47 Hz intermodulation frequency in the 300 ms-delay condition (*r* = 0.283, *p* = 0.255, BF_10_ = 0.532) and the 600 ms-delay condition (*r* =−0.076, *p* = 0.765, BF_10_ = 0.304).

## Discussion

In this study, we used EEG frequency tagging to examine the selective neural processing of visual and auditory information and their integration in the tracking of a moving stimulus and determine how spatial and temporal congruency between the two modalities modulate these mechanisms and the motion-tracking performance.

As expected, the results revealed significant EEG activity at both visual and auditory tagging frequencies (15 and 32 Hz, respectively) in both experiments. Activities at these frequencies were reliably observed in the EEG recordings of most participants in all conditions. These findings are in accordance with previous studies showing neural synchronisation to fast periodic visual and auditory stimuli in the form of SSEPs at the stimulation frequencies (Nozaradan et al. 2011, 2012, 2015; Aissani et al. 2011). Further, we observed stronger EEG responses at the visual frequency (*f*_V_) compared to the auditory frequency (*f*_A_). Although a number of factors might have influenced this difference, such as differences in how neural activity related to processing of the visual vs. auditory stimulus project onto the scalp and are captured with EEG or/and intensity of the stimuli, voluntary sustained attention towards the visual stimuli constrained by the visuomotor tracking task might have played a significant role. Participants were instructed to track the visual stimulus irrespective of the auditory information and sustained attention has been shown to increase stimulus-driven electrophysiological activity (Kim et al. 2007, 2017).

Spatial and temporal audiovisual (AV) congruency was expected to result in greater EEG response at the 15 and 32 Hz tagging frequencies, but no differences between Congruent and Incongruent conditions were found in any of the two experiments for any of the two frequencies. For the AV spatial congruency manipulation in Experiment 1, although not directly of interest for the current research, a larger response at the 32 Hz auditory frequency was found for the No motion condition compared to both Congruent and Incongruent conditions, which might be due to the constant intensity resulting in facilitated tracking and/or higher average loudness despite the same combined intensity across the two ears. We expected that incongruent auditory stimuli would generate conflict and elicit divided attention, whereas congruent auditory stimuli would increase attention towards the visual stimuli. Therefore, a smaller EEG response at the visual frequency was expected for the Incongruent compared to Congruent conditions due to divided attention (De Jong et al. 2010), which would also be associated to poorer motor tracking performance. However, we found no such effects in the SSEPs nor in the motor performance, contrary to our expectations and to previous research that showed enhanced SSEPs in temporally Congruent AV conditions compared to unimodal and Incongruent bimodal conditions (Nozaradan et al. 2012). Importantly, previous research showed that unimodal visual and auditory modalities differ on sensorimotor synchronisation (Hove et al. 2013; Varlet et al. 2012). Specifically, tapping synchronisation improved with visual and decreased with auditory continuous pacing signals, whereas discrete auditory signals improved sensorimotor synchronisation (Hove et al. 2013; Varlet et al. 2012). It has been further demonstrated that both the continuity of the pacer and whether the pacing and movement patterns match contribute to visuomotor synchronisation, but that such facilitation is greater influenced in visual continuous pacers in comparison to the discrete ones (Zelic et al. 2018). In turn, a similar setup investigating effects of the match between pacer and movement patterns on auditory–motor synchronisation indicated improved and less variable synchronisation for discrete pacers and continuous movement, and it was argued that due to lower spatial resolution of the auditory sensory modality the benefits of spatial pattern matching on auditory–motor synchronisation might be limited (Zelic et al. 2019). Therefore, it is possible that the absence of clear landmarks on the auditory loudness modulation in this study influenced auditory-motor synchronisation and might explain the null results irrespective of audiovisual congruence. Furthermore, in our experiment, participants were instructed to track the visual stimulus, which likely made them focus on the visual information disregarding the auditory one, which could have contributed to the null findings amongst conditions both on SSEPs and motor performance, and also to the larger peak at the visual 15 Hz frequency.

Also contrary to our initial hypothesis, in both experiments we found no significant EEG activity at the intermodulation frequencies (17 and 47 Hz), which were examined to investigate multisensory integration or segregation. No significant EEG activity at these frequencies was found in any participant or condition despite reliable responses at the fundamental frequencies. Despite non-significant effects at the behavioural level, activity at the intermodulation frequencies, at least for some of the 38 participants tested across the two experiments, could have been expected considering the large responses observed at both the visual and auditory tagging frequencies. While these results differ from those observed in previous unimodal studies, including in movement synchronisation tasks (Varlet et al. 2020), they corroborate the results of Giani et al. (2012), where intermodulation frequencies were not observed across visual and auditory stimuli in various experimental settings. Our study extends this research by showing that intermodulation frequencies across visual and auditory modalities do not necessarily occur in movement synchronisation tasks either.

Still, considering previous findings that successfully show audiovisual intermodulation frequencies (Regan et al. 1995; Drijvers et al. 2021), it is important to identify similarities and differences in the experimental designs to better understand the parameters that might facilitate audiovisual intermodulation frequency measurement to be achieved, along with further understanding of their functional meaning in multisensory integration. In Regan et al. (1995) audiovisual integration was identified while passively viewing and listening to stimuli presented at 10.304 and 5.054 Hz, respectively. It is noteworthy that participants were not engaged in any particular task whereas in our study participants were instructed to track the visual stimuli. Also, in Regan et al. (1995) the stimuli were stationary and the auditory stimuli were delivered only at the left ear, whereas in our study we presented horizontally moving visual and auditory stimuli and, further, the sound was presented binaurally. Therefore, it may be the case that AV integration is more prominent when participants are not tracking one specific modality from the two presented modalities, i.e. visual or auditory. Furthermore, one could also argue that lower frequencies may be more effective in generating AV integration detectable in intermodulation frequencies, since Regan et al. (1995) used lower tagging frequencies (10.304 and 5.054 Hz vs. 15 and 32 Hz in our study). However, such a simple low-pass filter effect is unlikely, since a recent study found AV integration evidenced by significant response at *f*_V_ − *f*_A_, using higher frequencies, specifically, *f*_V_ of 68 Hz and *f*_A_ of 61 Hz (Drijvers et al. 2021). Finally, the main focus of the study of Regan and colleagues (Regan et al. 1995) was to identify the spatial source in the brain supporting AV integration, the processing of *f*_A_ and *f*_V_ fundamentals, harmonics and intermodulation frequencies. There was no comparison of the different frequencies across conditions as tested in our study.

In turn, Drijvers et al. (2021) paradigm consisted in an active task where participants were presented with action verbs and gestures and had to identify which verb they heard in a forced-choice task. One important aspect to consider is that speech–gesture integration is an automatic process of extraction and combination of AV information to generate the percept of what the interlocutor intends to communicate (Soto-Faraco et al. 2004b). This process is so strong that mismatching facial and auditory information generates audiovisual illusions, such as the McGurk effect (McGurk and MacDonald 1976, see Marques et al. 2016 for a review). It is also noteworthy that in Drijvers et al. (2021) the auditory stimuli were modulated in amplitude, whereas we modulated the pitch, since sound amplitude modulation was used to generate the panning for the perception of sound movement*.* Finally, another relevant difference is that Drijvers and colleagues (2021) presented the stimuli with noise, a strategy that potentiates integration of both modalities to allow mutual disambiguation of speech and visual stimuli, whereas our stimuli were presented without noise. Therefore, these differential methodological approaches could have accounted for our lack of intermodulation findings.

Importantly, since no difference between congruent and incongruent auditory stimuli was found on behavioural synchronisation performance in the current study, it remains possible that different movement synchronisation tasks could have promoted audiovisual integration and significant activity at the intermodulation frequencies. The complexity of the stimulus trajectories used, and the task difficulty more generally, could have been insufficient to promote the integration and intermodulation frequencies. Considering differences with previous studies, another aspect to account for in future research is that our stimuli were neither ambiguous nor presented with noise. Adding such conditions could possibly foster integration, as the degradation of one modality has been shown to result in greater use of the other modalities (e.g. Drijvers et al. 2021; Parise and Ernst 2017; Stacey et al. 2020; Van der Zwan et al. 2009). Similar experiments could also compare shorter and longer trials, as SSEPs have been shown to be more pronounced for short trials (see Gander et al. 2010). More direct attention manipulations towards visual or auditory stimuli could also be tested. Importantly, the best parameters to promote integration across sensory modalities, and potentially the occurrence of intermodulation frequencies, remain unknown. There are numerous experimental manipulations that will have to be explored before confirming or excluding the relevance of intermodulation frequencies for measuring and understanding bimodal integration.

Moreover, we also found no significant correlation between the EEG power in the main or in the intermodulation frequencies and the behavioural responses in none of the conditions of the two experiments. This further confirms the absence of link between synchronisation performance and magnitude of EEG activity at the fundamental and intermodulation frequencies. This further supports the potential influence of focused attention on the visual stimuli, which was encouraged by the task instructions, in limiting or preventing the use of auditory information (De Jong et al. 2010; Kim et al. 2007). Conditions and instructions encouraging further focus on auditory information could have been more conductive for the occurrence of significant effects. Furthermore, in our experiment, none of the sensory modalities was degraded so participants could more easily rely solely on the visual information to perform the task (von Saldern and Noppeney 2013) while ignoring the non-target auditory modality. When visual and auditory information are discrepant during spatial localisation, and the visual stimulus is reliable (i.e. not degraded), the visual modality dominates via the “visual capture” of auditory information, and thus there is a distortion of the perceived auditory location (Neuhoff 2004). This is demonstrated in the classic ventriloquism illusion, where sounds seem to be displaced in the direction of a visual stimulus (Alais and Burr 2004). Moreover, both spatial and temporal factors play a significant role in this crossmodal effect, with auditory motion perception being strongly modulated by apparent visual motion (Soto-Faraco et al. 2004a, b). Such interpretation is also in line with Riels and colleagues’ (Riels et al. 2020) findings, where the only AV intermodal effect was observed during auditory target trials resulting in weaker visual SSEPs compared to auditory non-target trials, which argues in favour of selective attention models.

In this sense, the auditory stimuli in our experiment might have played as a distractor that participants attempted to ignore, as even the unimodal spatial detection of sound due to loudness modulation is not necessarily straightforward (Neuhoff 2004). However, we cannot be categorical in such interpretation, as we compared different conditions of AV input signals, but did not look into the processing of each unimodal stimulus separately. Examining the competing modalities combined and in isolation would be necessary to draw further conclusions (Gordon et al. 2019). Nevertheless, we are still scratching the surface when it comes to understanding the extent of application of EEG frequency tagging to detect multimodal integration and crossmodal interactions. The absence of EEG activity at the intermodulation frequencies in all participants and conditions in the current experiment remains rather surprising, and therefore encourages further investigations in multimodal settings in future research.

## Conclusion

To conclude, the current study provides no evidence of selective unimodal processing and bimodal integration of visual and auditory information using EEG frequency tagging in participants manually tracking a moving object. The absence of effects of spatial and temporal congruency manipulations at both behavioural and neural levels in the two experiments suggest that experimental conditions, such as task instructions and difficulty, as well as sensory signal degradations, might need to be further manipulated in future research to determine conditions in which bimodal information is beneficial for the motor tracking of moving objects in the environment. These null results also encourage further investigations in future studies of the relevance of EEG activity at the intermodulation frequencies while using frequency tagging for indexing and understanding multisensory integration processes (also see Adam et al. 2021).

## Data Availability

All data are held in a public repository, available at OSF database (URL access: https://osf.io/2jr48/?view_only=17e3f6f57651418c980832e00d818072).
